# Successful Removal of a Foreign Body from the Knee-joint

**Published:** 1852-09

**Authors:** J. Washington Smith

**Affiliations:** Croton, Delaware County, N. Y.


*Successful Removal of a Foreign Body from the Knee-joint.—Anomalous Character of the same.* By J. WASHINGTON SMITH, M. D., of Croton, Del. county, N. Y.

MESSRS. EDITORS,—The following case presents so many points of interest, if not of originality, in its history and treatment, that I have thought a record of it worth preserving—and, such as it is, I now place it at your disposal.

Mr. R. M., æt. 22, a laborer on a farm, otherwise healthy, consulted me in June, 1851, in regard to a difficulty of the right knee. For some months previous something had appeared to "catch," as he termed it, in the joint while using it, causing excruciating pain, followed by inflammation, swelling, and weakness of the joint. The peculiar "catch" had only existed for a few months, but the patient says, "the joint has been a little weak and swollen at times ever since a 'wrench' it received some four or five years since, during a wrestling feat." Recently he had perceived a "loose body, something like a bean," each side of the patella, and at other points, which was freely moveable and would easily slip into the joint. At times it was readily detected, at others difficult or impossible. The knee was considerably enlarged, as from chronic thickening of the synovial membrane, and tender to the touch, in places.

*Diagnosis.*—After the above history, and manipulating the joint, so as to bring the foreign body within reach, I did not hesitate to pronounce it a case of *loose cartilage* within the joint.

The probable cause, nature, course, usual treatment, &c., were fully explained, and the patient dismissed, with the advice not to think of an operation for its removal, unless the use and safety of the limb imperatively required it; and that efforts be made to "fix" or confine it *outside*, or else to prevent its escape from *within* the joint; also, to consult older and more experienced counsel.

At my request the patient called occasionally to report his condition, and enable me to watch the progress of the case. After a time he acquired such dexterity as to bring the foreign body outside of the joint almost at pleasure.

After September, 1851, he was not able to attend regularly to his occupation; and as the joint was becoming more thickened and stiffened, from the continued irritation to which it was exposed, efforts were made to confine the cartilage, (as I still supposed it,) by pushing it far away from the joint, in one of the synovial pouches by the side of the patella; but it was found, upon repeated trials, that it could not be retained *in situ* by any amount of pressure which could be borne, and allow any motion of the joint. Efforts were also made to *prevent* its escape from the joint, but with only partial success, while the cause of irritation was sure to be at work.

There now appeared but one other alternative—the chance of an operation—and which, owing to the state of the joint, it must be confessed was not very promising. The almost certain result of the case as it *was*, together with the *probable* and *possible* consequences of an operation for the removal of the offending body, were again fully and fairly stated to the patient and friends. Dr. Almiron Fitch, of Delhi, a distinguished member of the profession in this section of the State, was also consulted, and coincided fully in the opinion given.

*Operation.*—Early in March last I was requested to remove the foreign body by operation. Having enjoined rest, low diet, and occasional purgatives during the ensuing week, on the 10th instant I proceeded to remove it; Dr. Fitch kindly consenting to be present, and from whom I received valuable assistance. Some delay and difficulty were experienced in confining the same in the position desired, viz: upon the inner condyle, without occasioning too much motion of the joint. The integuments were then drawn tensely forward (about one inch) over the foreign body while the same was firmly fixed by the fingers of an assistant. A longitudinal incision, three-fourths of an inch in length, was then carried through the integuments directly upon the cartilage, (?) but, as it could not readily be removed, a second incision was made, so as slightly to enlarge the first, when with a tenaculum it was removed, with some difficulty, owing to the thickened state of the joint, and the unexpected *hardness* of the supposed cartilage. In form it much resembled an almond, was eight lines in length, six broad, and four in thickness. Its substance was evidently osseous or calculous, but unfortunately (and contrary to agreement) it passed out of my possession before I had an opportunity of subjecting it to any chemical or microscopic tests. It was completely enveloped in healthy looking cartilage.

The hemorrhage was slight, and soon ceased, when the incision was nicely secured by plasters, a compress carefully adjusted so as to securely close the *valvular* opening, and over these a figure of 8 bandage; the whole being completed by a long splint to outside of limb, secured by roller, except over the knee, nearly preventing all motion. Water was applied several times a day, and was the only application. There was a slight exudation of serum, but none of synovia. The diet was light for a few days, and small occasional doses of pil. cath. comp. and pulv. jalap. comp. were given, to procure the regular evacuation of the bowels. Union was partly by first intention, and partly by what Macartney calls the *modelling* process, *i. e.*, without suppuration. The case was closely watched, and there was no inflammation at any time.

After a few days the patient sat up part of the time, but the plasters and splint were not dispensed with until the thirteenth day, when the wound appeared perfectly united. A compress and figure 8 bandage were continued, and directions given to use and flex the limb but slightly; but from that time he began and continued to go about. April 15th there was only a slight weakness, and some stiffness upon flexing to an acute angle. At the end of six weeks he was attending to his ordinary occupation, and was able to join, as he was wont to do, in "the giddy mazes of the dance."

*Remarks.*—The operation by subcutaneous incision, as proposed by Prof. Syme and M. Goyraud, was hardly practicable, owing to the thickened state of the integuments, though it would in most cases greatly lessen the danger of inflammation. Wounds penetrating the cavities of joints, especially the larger, have ever been the dread of surgeons, and the common result an opprobrium to the healing art. This case is a striking illustration of the importance of *perfect rest* (of the joint) and of a *simple*, but not *too* antiphlogistic treatment; though in regard to the latter the previous habits should be our guide. Here there was evidently a *want* of action, though the diet was "light" for only a few days, but had it been more generous, I am satisfied complete union by first intention would have taken place—a most desirable result in wounds of joints.

The history of this case may, and will doubtless, satisfy some minds, that a *fragment* of bone was nearly or quite detached at the time of the injury mentioned; but to my mind it is not so conclusive. A section of one end of the foreign body revealed a hard, friable substance, more resembling calculus. But, was a calculus ever completely enveloped in healthy cartilage? If so, it is a most remarkable provision of nature to prevent injury to the joint. May not a peculiar abnormal state of the synovial membrane, from any cause, give rise to an adventitious growth or deposit in the synovia as well as in other fluids of the body? Loose cartilages and calculous concretions are not very usual, but I do not recollect any account of a foreign body in the cavity of a joint, similar to the present.

The patient consulted different surgeons, to whom he stated the symptoms and diagnosis; but it is a curious fact, that none of them, at the time, detected the foreign body, (though part were satisfied of its existence,) and at one time, believing it a case of chronic synovitis, counter irritants, and ung. iod. comp. were freely applied, to procure absorption!

